# The impact of iodinated contrast medium on bone mineral density (BMD) quantification in computed tomography: a monocentric retrospective study evaluating phases, sex and age differences

**DOI:** 10.1007/s11547-025-02030-x

**Published:** 2025-06-02

**Authors:** Philip Senti, Francesco Magoga, Oriana D’Ecclesiis, Andrea Cozzi, Filippo Del Grande, Rolf Wyttenbach, Stefania Rizzo

**Affiliations:** 1 Faculty of Biomedical Sciences, Università Della Svizzera italiana (USI), Via G. Buffi 13, 6904 Lugano, Switzerland; 2 Clinic of Radiology, Imaging Institute of Southern Switzerland (IIMSI), Ente Ospedaliero Cantonale (EOC), Via Tesserete 46, 6900 Lugano, Switzerland; 3 Independent Statistician, Rome, Italy

**Keywords:** BMD, CT, Opportunistic screening, Contrast medium

## Abstract

**Purpose:**

The primary purpose of this study was to assess the impact of iodinated contrast medium on bone mineral density (BMD) measurement in CT scans. The secondary purpose was to evaluate the impact of contrast medium on different acquisition phases, stratified by sex and age.

**Material and methods:**

CT examinations acquired in the emergency room between January 2019 and September 2021, before and after contrast medium administration, were included. On axial images, a round region of interest was traced on the first lumbar vertebra and the Hounsfield units (HU) values were recorded. Statistical analysis compared BMD differences across different acquisition phases. Stratified analyses by sex and age were also performed. Significance was set at *p* < 0.05.

**Results:**

A total of 295 CT acquisitions from 100 patients (52 males; 48 females) were included. There was a significant difference in BMD among native, arterial and portal venous phases (*p* < 0.001). Specifically, BMD in arterial and venous phases differed significantly from the native phase (p = 0.007 and *p* < 0.001, respectively). Males showed a BMD higher of 19.1 points compared to females (*p* = 0.0007); younger people showed a higher BMD compared to older people (*p* < 0.001). Overall, significant differences in density emerged between phases in all stratified analyses.

**Conclusion:**

Unenhanced and enhanced CT shows significant differences in BMD quantification, particularly when comparing the venous and native phases. These differences were consistent across analyses performed according to sex and age.

## Introduction

Osteoporosis is characterized by a reduction in bone mass, leading to increased risk of fractures and mortality [1]. It has become a significant public health concern due to its high prevalence and substantial economic burden [2, 3]. In the Caucasian population of the USA, approximately one in two women and one in five men aged 50 years and older will experience an osteoporotic fracture in their remaining lifetime [4]. If these fragility fractures lead to complications, they result in a considerable increase in morbidity and mortality, with a one-year mortality rate of up to 30% following an osteoporotic hip fracture [5].

Currently, the diagnosis of osteopenia and osteoporosis, as well as the prediction of fracture risk, relies on bone mineral density (BMD) measurements obtained via dual-energy x-ray absorptiometry (DXA) [6, 7]. However, DXA results can be affected by patient positioning errors, artifacts like implants or fractures, and incorrect region selection, leading to inaccurate BMD readings. Calibration inconsistencies and operator variations further impact results, potentially misclassifying osteoporosis severity and affecting treatment decisions [8].

Some studies have demonstrated that computed tomography (CT) may assess BMD more sensitively than DXA due to its ability to directly measure trabecular bone density[9]. However, CT has higher costs, lower availability and delivers a higher ionizing radiation dose compared to DXA [10], which typically emits only 1–10 microsieverts (µSv), a dose comparable to natural background radiation [11]; therefore, this technique cannot replace DXA as primary diagnostic imaging. Despite these limitations, the opportunistic use of CT scans as screening tool for osteoporosis may be beneficial for patients who routinely undergo CT scans and are at risk of BMD loss, such as oncological patients, patients with chronic illnesses and patients under long-term medications [12–17].

To support the use of CT scans for BMD assessment, reference BMD values in Hounsfield units (HU) for different age and sex ranges derived from non-contrast-enhanced CT scans have been proposed [18]. The effectiveness of non-contrast-enhanced CT for this purpose has been further confirmed by additional studies [12, 19]. However, these findings cannot be directly applied to CT scans acquired after contrast medium injection, which unfortunately excludes a significant number of CT scans from being used for opportunistic BMD screening [12–17, 20].

Therefore, the primary purpose of this study was to build upon existing research by retrospectively assessing whether the injection of contrast medium significantly alters BMD measurements in patients who underwent CT scans before and after the intravenous administration of iodinated contrast medium, in multiple phases. By further investigating this controversial topic, this study aims to provide additional insights and clarify the extent of contrast medium impact on BMD assessments.

The secondary purpose was to evaluate the impact of contrast medium administration on BMD across different post-contrast phases, considering sex and age.

## Material and methods

This study was approved by the local ethics committee (protocol code 2021-00943). A non-objection letter was sent to all patients who had not previously provided a general consent for the use of their coded data for research purposes.

*Patient and CT selection* Inclusion criteria were as follows: inpatients and outpatients who underwent a CT examination after admission to the emergency room at our Institution in Ticino (Switzerland) between January 2019 and September 2021 for various clinical indications with at least two CT phase acquisitions available (see dedicated section). Exclusion criteria were as follows: known malignancy; fractures of the L1 vertebra or the presence of artifacts from metallic devices that could impair bone density measurement [21]; and age < 18 years.

*CT examinations* CT examinations included in this study were performed on one of the six CT scanners available at our institution (1 Somatom Definition Flash, Siemens Healthineers, Erlangen, Germany; 4 Somatom Definition Edge, Siemens Healthineers, Erlangen, Germany; 1 Brilliance ICT, Philips Healthcare, Eindhoven, Netherlands). All CT scans included at least one acquisition before (native phase) and one acquisition after the administration of iodinated contrast medium (portal venous phase). If other acquisition phases after contrast medium administration were present (arterial phase and delayed phase), they were also recorded. The contrast medium administered during the selected period was always Accupaque 350 (GE Healthcare, Switzerland); the injection rate of the contrast medium was selected according to the clinical indication (e.g., in the suspect of a bleeding, an arterial phase was acquired with high injection rate [22]; in case of hematuria, a delayed phase was acquired [23]). The amount of contrast medium injected took into account the patient’s weight [24], according to the following formula: patient weight × 1.42 (converting factor for iodinated contrast media at a concentration of 350 mg/kg). The peak voltage depended on the body size, as per dose savings in routine clinical acquisitions.

**Fig. 1:**
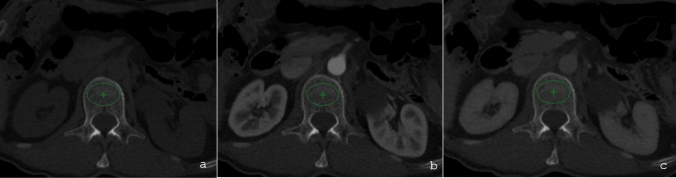
Example of three axial CT images at the level of the first lumbar vertebra acquired in the pre-contrast (**a**), arterial (**b**) and portal venous (**c**) phases. A region of interest containing trabecular bone (excluding cortical bone) was traced

*BMD measurement* On axial CT images with slice thickness of 3 mm, a region of interest (ROI) of 350-450mm^2^ which strictly contained trabecular bone and excluded cortical bone was traced at the level of the first lumbar vertebra (L1), and the mean and standard deviation of HU values were recorded (Fig. 1). As suggested by existing literature, we selected L1 because it is easily recognized as the first non-rib bearing vertebra and is generally less angled to the axial plane compared with L5 [25]. The analysis was performed by a single reader (P.S.), without the copy–paste tool, because positioning might have been slightly different across different phases according to subtle movements of the patients. Consistency was maintained through comprehensive training and the application of strict exclusion criteria, ensuring that diseased or partially fractured vertebrae were excluded (as mentioned in exclusion criteria).

### Statistical analysis

Median and interquartile range (IQR) for continuous variables and absolute and relative frequencies as summary measures of categorical variables were calculated. Friedman's test was used to test the statistical difference of density in different acquisition phases. Pairwise Wilcoxon rank sum test with a Bonferroni correction was used to specifically identify between which phases there was a statistically significant difference. For secondary purposes, analysis stratified by sex and age was also performed. The stratified analyses also used the pairwise Wilcoxon rank sum test with a Bonferroni correction. As a measure of the effect, Cliff's delta was used to express in numerical terms the intensity of the difference between the phases with respect to the outcome. A nonlinear mixed-effects model was applied by adjusting for age, sex and stages as fixed-effects variables while considering patient as a random effect. Boxplots were generated for the graphical representations, and all p-values were two-sided with a 5% significance level. The statistical analysis was carried out using the R studio (R version 4.3.2) software.

## Results

According to the inclusion and exclusion criteria, our study cohort comprised 295 CT acquisitions performed on 100 patients (52 males and 48 females) with a median age of 77 years (IQR 66.7–82.0). Native and venous phase was acquired for all patients (n = 100); the arterial phase was acquired for 95 patients; delayed phase was acquired for 22 patients, which was therefore excluded from further analyses. Injection rate of contrast medium ranged between 2.5 and 4.5 ml/s, depending on the clinical indication for the CT scan. The peak voltage ranged between 90 and 120 kV according to the body size.

**Fig. 2:**
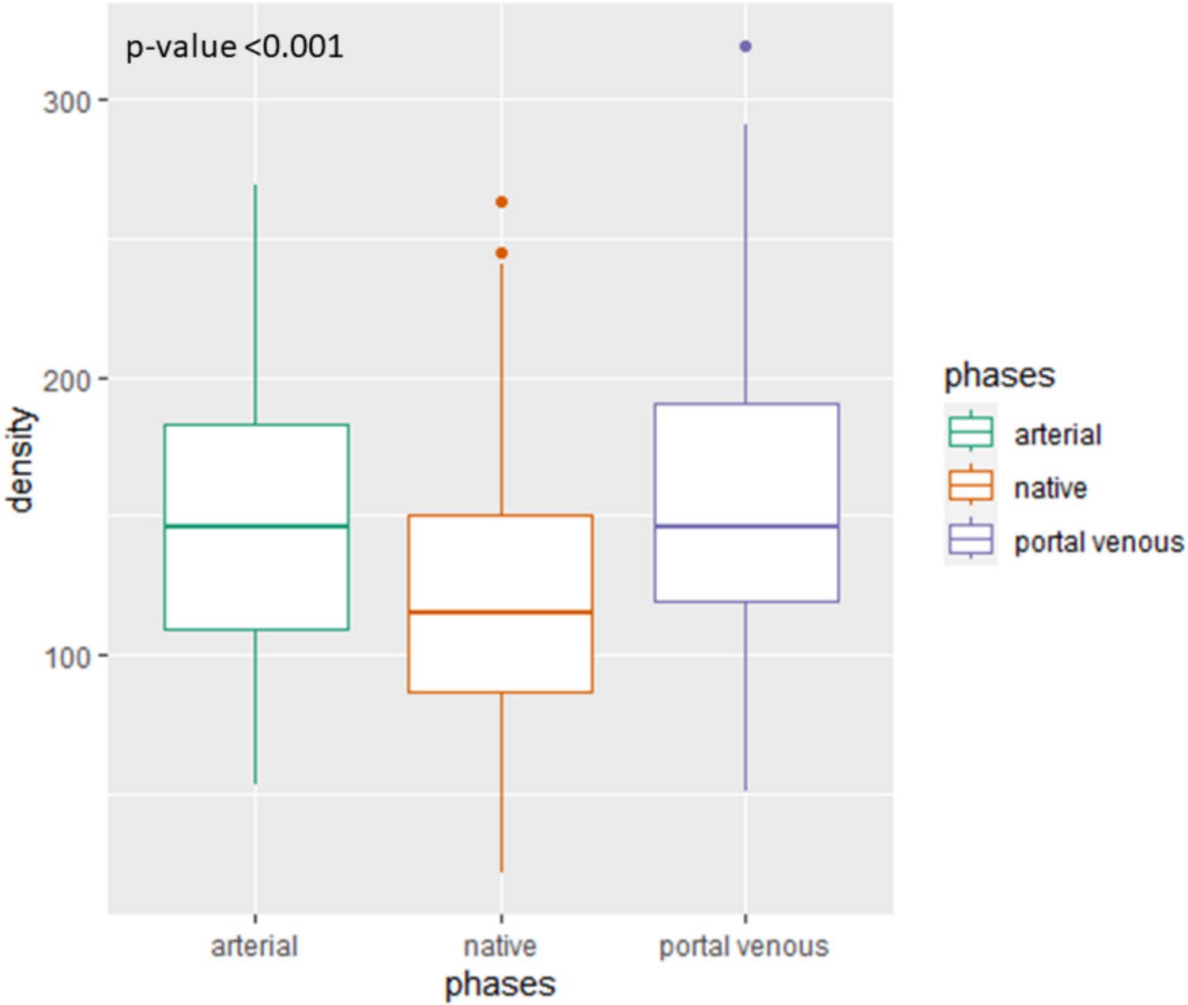
Boxplot showing density values in different acquisition phases: arterial (green) and portal venous (blue) compared to the native phase (orange)

As shown in Fig. 2, boxplots of density demonstrated a significant difference in density across the native, arterial and portal venous phases (*p* < 0.001). The pairwise comparison of densities (HU) between the three phases revealed that both the arterial and venous phases differed significantly from the native phase (*p* = 0.007 and *p* < 0.001, respectively), whereas no significant difference was observed between the arterial and venous phases (*p* = 0.99, Table 1). Further analysis, as shown in Table 2, using a mixed model with patients as random effects and age, acquisition phase and sex as fixed effects, revealed that the venous phase had on average an increase of 28.8 points on density, compared to the native phase (*p* < 0.001). The arterial phase had on average a density higher of 22.3 points compared to the native phase (*p* < 0.001).

**Table 1:** Comparison of bone mineral density (HU) across different CT phases of acquisition

	Arterial	Native
Native	**0.007**	–
Venous	0.99	** < 0.001**

*p* < 0.05 in bold

**Table 2:** Mixed model analysis with patients as random effects and age, acquisition phase and sex as fixed effects

Variable	HU estimate (SE), p-value
*Phases*	
Arterial vs native Portal venous vs native	22.3 (2.09), < 0.001 ^*^ 28.8 (1.88), < 0.001 ^*^
*Sex*	
Male vs female	19.1 (7.04), 0.007 ^*^
*Age*	
< 77 vs ≥ 77	36.8 (7.05), < 0.001 ^*^

HU = Hounsfield units

^*^ Significant *p*-values

Males, on average, showed a density higher of 19.1 points compared to females (*p* = 0.0007). Younger individuals (below the median age) exhibited higher density than older individuals (above the median age) (*p* < 0.001; Table 2). Even not excluding the delayed phase from the model does not change the results.

**Fig. 3:**
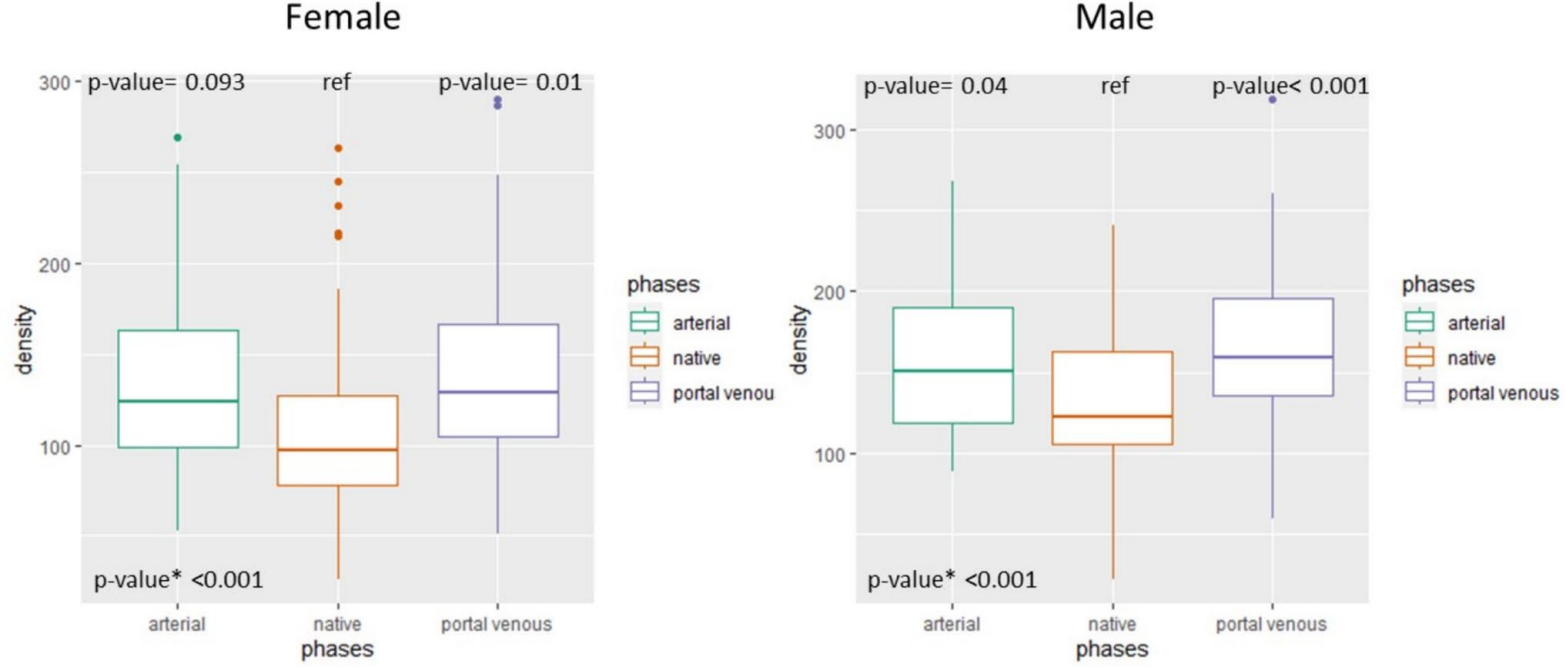
Boxplot showing density values in different acquisition phases, stratified by sex. p* indicates the p-value comparing all three phases together

The median and IQR HU values after splitting the cohort according to sex are shown in Table 3. As shown in Fig. 3, there was a significant difference in density when comparing all phases for both sexes (*p* < 0.001). Pairwise comparisons of density between acquisition phases showed a significant difference between the portal venous and native phases for both males (*p* < 0.001) and females (*p* < 0.01). However, the comparison between the arterial and native phases showed a significant difference only for males (*p* = 0.04) and not for females (*p* = 0.093).

**Table 3:** Median (IQR) density values for each phase, stratified by sex

Sex	Native HU, median (IQR)	Arterioso HU, median (IQR)	Venoso HU, median (IQR)	Delayed HU, median (IQR)
Female	97.0 (78.2–126.9)	124.0 (99.1–163.5)	129.3 (104.7–166.6)	128.4 (95.7–141.6)
Male	122.1 (105.1–163.2)	150.7 (118.3–190.1)	159.0 (135.6–196.2)	134.0 (107.8–196.5)

**Fig. 4:**
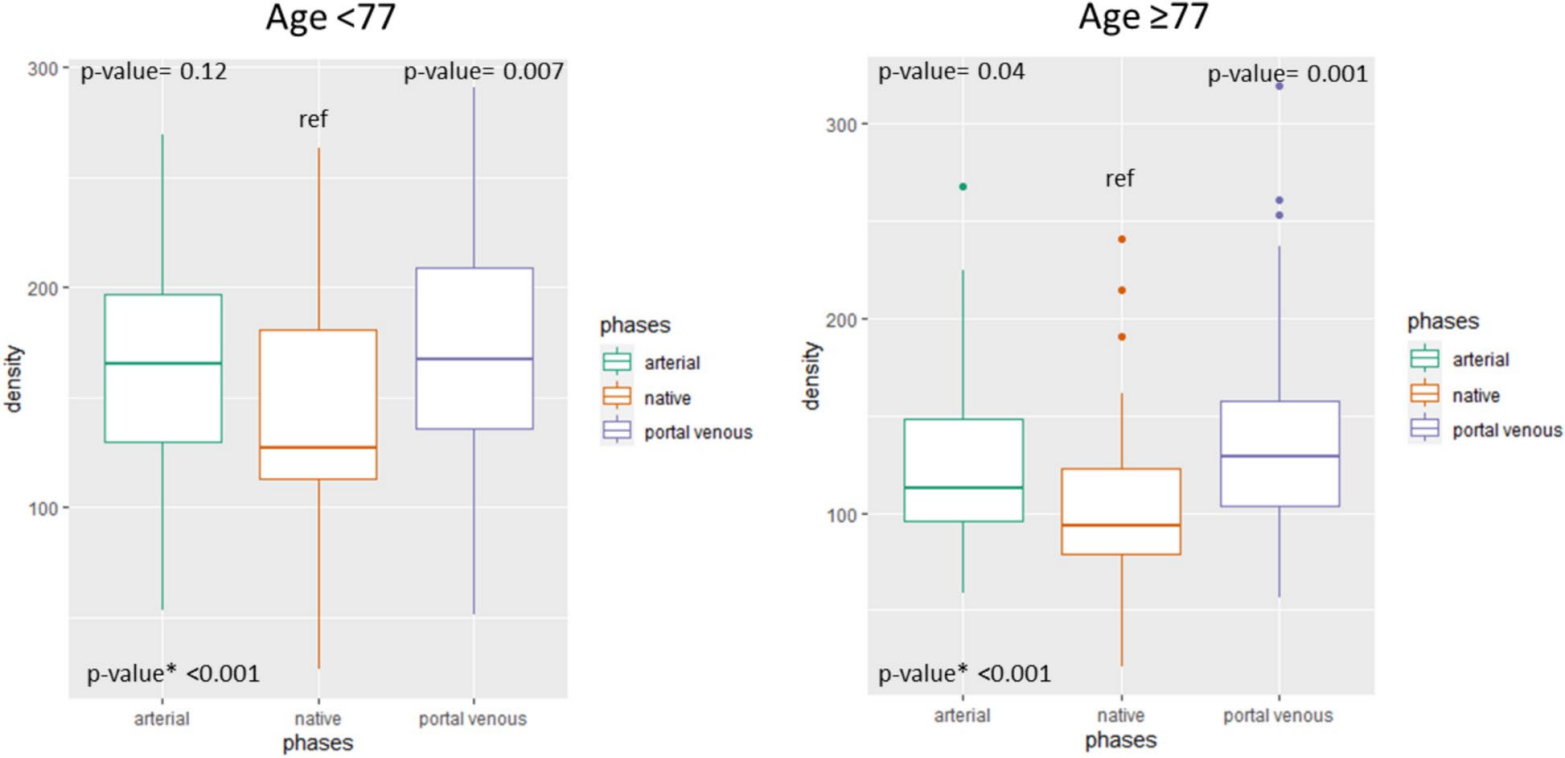
Boxplot of density values in different acquisition phases, stratified by age. p* represents the *p*-value comparing all three phases together

The median and IQR HU after splitting the cohort according to age are presented in Table 4. As shown in Fig. 4, there was a significant difference in density comparing all phases (*p* < 0.001) for both age groups. The difference in density was significant when comparing the portal venous phase with the native phase for both age groups (for age < 77 *p* = 0.007; for age ≥ 77 *p* = 0.01). However, the difference between the arterial and native phases was significant only for those aged ≥ 77 (*p* = 0.04) and not for those aged < 77 (*p* = 0.12).

**Table 4:** Median (IQR) density values for each phase, stratified by age

Age	Native phase HU, median (IQR)	Arterial phase HU, median (IQR)	Venous phase HU, median (IQR)	Delayed HU, median (IQR)
< 77	127.2 (112.6–180.5)	165.0 (129.4–197.0)	167.2 (135.9–209.1)	154.3 (127.6–177.4)
≥ 77	93.4 (79.2–122.7)	113.0 (96.1–148.4)	128.7 (103.9–157.6)	107.2 (93.7–136.5)

Table 5 shows the Cliff's delta values between the phases; the results are consistent with previous analyses.

**Table 5:** Cliff's delta between phases

	Arterial	Native	Delayed
*Overall*			
Native	0.28	–	0.22
Venous	− 0.08	** − 0.36**	− 0.17
Delayed	0.09	− 0.22	–
Arterial	–	− 0.28	− 0.09
By sex			
*Female*			
Native	0.29	–	0.32
Venous	− 0.06	** − 0.35**	− 0.10
Delayed	0.07	− 0.32	–
Arterial	**–**	− 0.29	− 0.07
*Male*			
Native	0.31	–	0.17
Venous	− 0.14	** − 0.42**	− 0.24
Delayed	0.10	− 0.17	–
Arterial	**–**	** − 0.31**	− 0.10
*By age* *** < 77***			
Native	0.27	**–**	0.24
Venous	− 0.11	** − 0.37**	− 0.23
Delayed	0.12	− 0.24	–
Arterial	–	− 0.27	− 0.12
* ≥ 77*			
Native	0.32	–	0.28
Venous	− 0.11	** − 0.41**	− 0.17
Delayed	0.08	− 0.28	–
Arterial	–	** − 0.32**	− 0.08

## Discussion

In this study, we confirm that the injection of iodinated contrast medium significantly affects BMD values on CT images. This finding aligns with previous studies that have reported similar results [26–33]. Our study specifically demonstrates these effects in both the arterial and venous phases compared to unenhanced images, additionally revealing significant differences based on sex and age through a dedicated analysis including these two factors.

Our results show that BMD measurements after the administration of contrast medium remain consistent across both sexes and all age groups in the portal venous phase, compared to the native phase. Given these inhomogeneous results, implementing CT scans for opportunistic BMD screening in clinical practice would benefit from the inclusion of the same acquisition phase to ensure consistency and enable reliable comparisons. For example, CT examinations for cancer monitoring almost always include the portal venous phase but do not always include the native phase [26, 34]. A study set HU reference values for L1 in osteopenia (< 106.9) and osteoporosis (< 162.4)[35]. Another one proposed HU cutoff values for diagnosing osteoporosis of 89.7 in the native phase and of 96.7 in the cortico-medullar phase [36–38]. In our study, we found an average increase of 28.8 points in the venous phase compared to the native phase, meaning that if no standardization or conversion factors would be implemented, patients with only a venous phase available on average would not be detected with osteoporosis until they have a value of 78.1 HU, which is well below the abovementioned cutoffs.

Our study suggests that CT scans can be used to assess BMD, though careful selection of the analyzed phase is necessary. Ultimately, when selecting the same acquisition phase, comparisons and evaluations of BMD on CT scans can be reliable and consistent over time.

The possibility of mining additional information retrospectively from radiological images for opportunistic screenings has gained growing interest. Diagnostic examinations acquired for one clinical indication may provide valuable insights into other health conditions, including body composition [39] and bone density status [18, 25]. Opportunistic screenings could be particularly beneficial for individuals not enrolled in organized screening programs [19], as well as for patients who regularly undergo CT scans and also require DXA assessments due to their high risk of developing BMD loss, such as cancer patients and those on long-term medications affecting BMD [15, 40]. In these cases, quantitative BMD measures from CT scans could help identify early signs of osteoporosis and guide management with interventions like calcium supplementation, vitamin D, or bisphosphonates to reduce fracture risk. The possibility of pre-symptomatic osteoporosis detection through opportunistic screening has been demonstrated [40]. Some authors have shown that fully automated CT-based BMD measurement of L1 attenuation correlates positively with the Fracture Risk Assessment Tool and accurately predicts future fragility fractures [12, 19].

Despite its enormous clinical potential, a systematic review found that direct HU measurement from diagnostic CT scans is not yet suitable for routine clinical use [27]. The use of CT for osteoporosis evaluation remains debated and under-implemented, likely due to inconsistencies across studies, including differences in BMD measurement sites, CT machines, protocols, and contrast medium administration [27]. A systematic review of 16 studies showed that contrast medium increases BMD measurements, with one study reporting a 7.5 HU increase in bone density on contrast-enhanced CT scans compared to non-contrast scans (*p* < 0.01)) [28]. Another study, including 1187 subjects, demonstrated an average increase of 26.7 HU in contrast-enhanced portal venous phase CT images (*p* < 0.001) [26]. These findings align with other studies showing higher BMD values in contrast-enhanced CT scans[29–32], with one study suggesting that 25% of participants may be reclassified from osteoporotic to non-osteoporotic after contrast administration [30]. In cardiac CT, contrast medium led to misclassification of BMD in 24% of cases, with 6% misclassified as having low BMD despite very low BMD on unenhanced images, highlighting significant overestimation of BMD in contrast-enhanced images [33]. These misclassifications can affect the clinical accuracy of fracture risk assessments, such as those based on the FRAX score.

Some studies reported no significant differences in volumetric trabecular BMD values based on contrast medium presence, whether using dual-source dual-energy CT [41] or single-energy multidetector CT scanners [37]. A study of 157 patients showed an 11 HU increase in enhanced over unenhanced L1 trabecular attenuation (*p* = 0.632), concluding that both enhanced and unenhanced scans can be used for initial osteoporosis screening via CT [42]. Another study involving over 20,000 adults found a significant difference in HU values with and without contrast only in patients younger than 40 years [38], suggesting that contrast medium may not impact screening for older patients. The possibility of calculating a conversion factor to transform CT-derived BMD values into diagnostically relevant values has been explored in quantitative CT [31], as well as in dual-layer CT [43]. Establishing such a conversion factor remains challenging, but its introduction could greatly enhance opportunistic osteoporosis screening, facilitating the integration of retrospective CT scans into daily clinical practice and leading to an earlier access to relevant preventive care.

In the future, fully automated CT-based BMD measurements, which account for and correct the differences between contrast-enhanced CT and non-contrast equivalents, might facilitate the evaluation of mixed CT cohorts, thus expanding the potential for individualized opportunistic screening [42]. A promising experimental study using photon-counting detector CT on Sprague–Dawley rats demonstrated that photon-counting CT was effective in characterizing bone conditions. This was tested using hydroxyapatite phantoms (*n* = 6) with varying calcium concentrations at selected energy bins [44], highlighting the potential future applications of this technology for BMD assessment.

Many societies encourage sex-related analyses because of the intrinsic physical differences between males and females. The effect of aging on bones includes trabecular thinning and loss of trabecular connectivity, and the rate of these processes differs between men and women. Sex hormones may influence maximum bone mass and architecture. Indeed, osteoclasts own estrogen receptors but no androgen receptors. Women commonly develop a rapid decrease in estrogen levels with menopause, as compared with men after andropause. As a consequence, menopause is associated with a 90% increase in bone resorption but only a 45% increase in bone formation, leading to an acceleration of bone loss and to its related higher fracture risk [45, 46]. In our study, we observed a significant difference of BMD in both sexes between the portal venous and native phases. Considering that sex can influence BMD measurements, tailoring more individualized treatment plans could be beneficial. For example, a female cancer patient may show lower BMD in the native phase, but the administration of contrast medium may mask this reduction, leading to a falsely elevated BMD reading. This highlights the need for gender-adjusted BMD values when using CT for osteoporosis screening.

The analyses performed according to age confirmed that aging is associated with bone loss [38], with a physiological reduction in BMD in older individuals (> 77 years). For this reason, when elderly patients undergo CT scans, such as those for cardiovascular disease or abdominal imaging, they may benefit from opportunistic osteoporosis screening. Furthermore, other high-risk factors should be included in further analyses, accounting for chronic illnesses and long-term medications, particularly those that are known to affect bone density (e.g., glucocorticoids, aromatase inhibitors and anticonvulsants). A recent analysis performed by Medicare on the economic value of BMD evaluation as opportunistic screening demonstrated that if only non-contrast CT was used to identify osteoporosis and treatment was successfully implemented in 100% of eligible beneficiaries, this study population would see a medical cost avoidance in excess of $17 million; furthermore, if any CT was used, disregarding the presence of contrast medium, potential annual cost avoidance for the same population would be nearly $100 million and $2.5 billion for all 2023 Medicare fee-for-service beneficiaries [47].

Our study contributes interesting insights to this topic by confirming the significance of factors such as contrast medium phases and highlighting the importance of sex and age.

This study has some limitations. First, we did not include acquisition parameters such as tube current and voltage, contrast dose and injection rate which may affect the HU values. However, all CT examinations were performed at the same institution, where the acquisition protocols were standardized based on clinical indications, and the acquisition parameters were kept constant across all CT scanners. Furthermore, during the selected period, only one iodinated contrast medium was used at all sites, ensuring a constant iodine concentration in the injections. Other parameters such as comorbidities of the patients were also not included in our analysis even though comorbidities can influence bone mineral density (BMD), as our primary focus was to examine the effect of contrast medium on BMD measurements, rather than the impact of comorbidities. Further studies, including more details about the comorbidities of patients are warranted to evaluate different clinical settings and perhaps to establish different cutoffs according to clinical conditions. Second, we employed a simple method to assess BMD on axial CT images, based on a circular ROI traced by a single reader, whereas some AI-based tool for these evaluations may be available (mostly for research use). However, incorporating AI for BMD evaluation on CT scans was beyond the scope of this study and would require a dedicated investigation. Lastly, we were unable to assess whether the differences in HU across different contrast phases were related to the abundance of bone marrow. Currently, no tool exists that can distinguish between red marrow, white marrow and the trabecular components of bone. In the future, if such tool is developed, BMD assessment based on CT density could be more accurately evaluated and monitored over time, regardless of contrast medium presence or phase. In conclusion, our study confirms significant differences in BMD quantification on CT before and after intravenous administration of contrast medium in both arterial and venous phases, with variations across sex and age groups. Maintaining consistency in the choice of acquisition phases for BMD assessment in CT-based opportunistic screenings is crucial, as it may avoid misleading conclusions regarding fracture risk, which may arise from technical quantification factors, rather than actual differences in BMD. While these findings highlight the potential of CT scans for opportunistic osteoporosis screening, caution is needed in clinical applications until HU standardized correction models and as phase-specific thresholds are developed, or vendor-neutral algorithms are implemented as clinical tools.

In this study, we demonstrated that the presence of iodinated contrast medium significantly affects BMD values on CT images, with higher values in post-contrast images compared to non-contrast images. Furthermore, these differences were consistent across sex and ages.

Future research should focus on developing phase-, sex- and age-specific correction models to standardize BMD measurements across different contrast phases, possibly integrating AI-driven algorithms for automated phase recognition and quantification, and validating CT-derived BMD values against DXA, which is the current clinical gold standard. By refining these methodologies and properly analyzing bone density as opportunistic evaluation in CT scans, clinicians could earlier detect signs of osteoporosis, especially in patients who might otherwise not undergo dedicated screening, potentially improving patient outcomes through timely intervention.

## Data Availability

No datasets were generated or analyzed during the current study.

## Abbreviations

BMD: Bone mineral density
DXA: Dual-energy x-ray absorptiometry
HU: Hounsfield units
IQR: Interquartile range
L1: First lumbar vertebra
ROI: Region of interest
